# Tomato activates ethylene signaling to maintain *pathogenesis-related genes* expression for conferring bacterial wilt resistance

**DOI:** 10.3389/fpls.2025.1753391

**Published:** 2026-02-16

**Authors:** Weiwei Cai, Qiquan Li, Feina Liu, Yilin Tao, Zhen Xu, Zhujun Zhu, Yuan Cheng, Li Tian

**Affiliations:** 1 Key Laboratory of Quality and Safety Control for Subtropical Fruit and Vegetable, Ministry of Agriculture and Rural Affairs, Collaborative Innovation Center for Efficient and Green Production of Agriculture in Mountainous Areas of Zhejiang Province, College of Horticulture Science, Zhejiang Agriculture and Forestry University, Hangzhou, Zhejiang, China; 2 Vegetable Research Institute, Zhejiang Academy of Agricultural Sciences, Hangzhou, Zhejiang, China

**Keywords:** ethylene, PR genes, Ralstonia solanacearum, RNA-Seq, tomato

## Abstract

Bacterial wilt that caused by *Ralstonia solanacearum* poses a major threat to tomatoes. Some disease-resistant cultivars have been shown to significantly improve tomato resistance to bacterial wilt. Analyzing and harnessing the resistance mechanism of bacterial wilt-resistant cultivars is therefore of considerable importance for tomato resistance breeding. In this study, we have confirmed that the tomato disease-resistant cultivars “ZJ-7” and “04056” were more resistant to bacterial wilt than the susceptible cultivar Ailsa Craig cv. (AC), and then transcriptome sequencing analysis of roots from “ZJ-7” and “04056” revealed extensive changes in gene expression at 3 and 6 hours post-inoculation (hpi) with *R. solanacearum*. In both disease-resistant cultivars, the transcriptional expression levels of genes encoding pathogenesis-related (PR) proteins and transcription factors were markedly elevated in response to *R. solanacearum* infection at both time points. In contrast, the susceptible cultivar AC exhibited a considerably lower number of transcription factors responding to the infection, with up-regulated occuring only at 6 hpi, while the up-regulation of *PR* gene expression was observed only at 3 hpi. Although the specific up-regulated genes differed between “ZJ-7” and “04056”, both showed activation of ethylene biosynthesis-related genes. Ethephon application in AC promoted the expression of transcription factors at 3 hpi and restore PR gene expression at 6 hpi. These results indicate that sustained defense against bacterial wilt in tomato is closely assocated with ethylene synthesis, providing a theoretical basis for elucidating the resistance mechanism and enhancing disease resistance in tomato.

## Introduction

Tomato (*Solanum lycopersicum*) is one of the important vegetable crops worldwide, with high nutrition and unique flavor, ranking first in vegetable production (Zhang et al., 2024). Native to South America, tomatoes thrive in warm and humid controlled environment such as greenhouses (Sato et al., 2012). However, due to climate change, tomato diseases are becoming increasingly serious (Meline et al., 2022), among which bacterial wilt, caused by *Ralstonia solanacearum* (*R. solanacearum*) species complex (RSSC) (Vailleau and Genin, 2023), is particularly devastating. This pathogen complex collectively infects the vascular bundles of tomato roots from the soil synergistically, blocking water transport and ultimately causing plant death.

To mitigate the damage caused by RSSC, several control strategies have been developed. These include modifying soil microbial communities, improving drainage systems, applying bactericides, and most widely adopted-grafting techniques (Yuliar et al., 2015; Jiang et al., 2025). Employing rootstocks with strong resistance has proven highly effective in preventing bacterial wilt in tomatoes. However, grafting involves considerable labour costs and often results in the loss of some seedlings (M J Asins et al., 2015; Aphrodite et al., 2021; Zhou et al., 2022). Moreover, recent studies indicate that rootstock selection can influence fruit quality in the scion (Zhou et al., 2022; Gong et al., 2023). Therefore, elucidating the mechanisms underlying bacterial wilt resistance in tomato rootstocks and leveraging this knowledge for genetic improvement represent a crucial strategy for combating the damage inflicted by this disease.

Currently, the defense mechanisms of tomatoes against RSSC infection have been preliminarily elucidated (Xue et al., 2025; Chachar et al., 2025). Most terrestrial higher plants, including tomatoes, lack specialized immune organs. and rely on cellular-level defenses, forming two distinct layers of immune barrier, Pathogen Associated Molecular Pattern (PAMP)-triggered Immunity (PTI) and Effector-Triggered Immunity (ETI) (Yu et al., 2024). In *Arabidopsis thaliana*, the elongation factor EF-TU and flg22 (a conserved fragment of bacterial flagellin) are recognised as PAMPs Microbe-Associated Molecular Patterns (MAMPs) by the the pattern recognition receptors (PRRs) FLS2 and EFR, respectively, thereby activating PTI against R. *solanacearum* (Macho et al., 2014; Boschi et al., 2017; Li et al., 2025). However, in Solanaceae plants such as tomatoes, the absence of EFR and the ability of *R. solanacearum*-derived flg22 to evade FLS2 recognition impede the immune recognition of the pathogen. Exogenous expression of EFR or modification of the flg22 enables tomatoes to recognize *R. solanacearum*, thereby enhancing their resistance. In tomatoes, cold shock protein 22 (CSP22) serves as the key molecule for recognizing *R. solanacearum* and activating PTI. It is specifically captured by the tomato csp22 receptor (SlCORE), and the immune signal is transduced downstream via tomato csp22-activated kinase 1 (SlCAK1) (Wei et al., 2017; Zeiss et al., 2022; Li P. et al., 2025).

As a conserved basal immune mechanism, PTI is often targeted and disrupted by effector proteins evolved by pathogens. Effector are a class of secreted proteins synthesized by pathogens that function within host plant cells. More than 100 different Type III effectors have been identified in *R. solanacearum* (Puigvert et al., 2018). These effectors can mimic various intracellular plant proteins like proteases to interfere with normal immune signalling cascades (Qi et al., 2022; Choi et al., 2025). For instance, the effector RipE1 acts as a cysteine protease that cleaves the plant immunity regulator SlRIN4, thereby suppressing host defences (Lu et al., 2024). Nucleotide-binding site-leucine-rich repeat (NLR-LRR) proteins (NLRs) typically contain NBS-LRR domains and recognize effector proteins either directly or indirectly, triggering ETI response (Cui et al., 2015; Yuan et al., 2021; Yu et al., 2024). For example, SlADR1 and SlNRG1 both contribute to the immune function of the ‘Hawaii 7996’ tomato cultivar against the pathogen (Xu et al., 2023). Collectively, ETI and PTI are closely interconnected, forming the molecular basis that enables plants to recognize pathogens and activate their immune responses.

Notably, neither PRRs nor NLRs directly engage pathogens. Instead, upon sensing pathogen invasion, they transmit immune signals from the cell membrane or cytoplasm to the nucleus (Ngou et al., 2021). This signal undergoes amplification in the nucleus and act on transcription factors to trigger extensive transcriptional reprogramming. This reprogramming activates a cascade of downstream immune responses, including reactive oxygen species (ROS) bursts, mitogen-activated protein kinase (MAPK) signalling pathway activation, cell wall reinforcement and upregulation of defence-related genes (Caplan et al., 2008; Moore et al., 2011; Ngou et al., 2021). Transcription factors play a pivotal role in receiving disease resistance signals and regulating downstream defence gene expression (Campos et al., 2022). For instance, the NAC transcription factor SlNAP1 promotes salicylic acid (SA) synthesis by activating *SlPAL3*, while the WRKY transcription factors SlWRKY30 and SlWRKY81 cooperatively enhance *SlPR-STH2* expression (Wang et al., 2020; Dang et al., 2023). Core resistance-related genes primarily include those invovled in defence response, signal transduction and secondary metabolism. Among defence-related proteins, Pathogenesis-related (PR) proteins, chitinases and protease inhibitors directly inhibit pathogen growth or degrade their cell walls (Dos Santos and Franco, 2023), with PR proteins being the most representative (Dos Santos and Franco, 2023). For instance, SlPR3 and SlPR-STH2 expression is significantly upregulated in response to RSSC infection, directly inhibiting pathogen proliferation (Dos Santos and Franco, 2023; Dang et al., 2023; Li P. et al., 2025).

Plant hormones signalling pathways also play critical roles in plant immune signal transduction. Despite differences in amplitudes, timings and robustness, PTI and ETI share common signalling pathways mediated by SA and jasmonic acid (JA) (Saleem et al., 2021; Peng et al., 2021; Ding et al., 2022; Ngou et al., 2022). SA is a crucial hormone in defending against biotrophic and hemibiotrophic pathogens, and mediates PTI, ETI and systemic-acquired resistance, while JA primarily defends against phytophagous insects and necrotrophic pathogens (Yu et al., 2024). SA and JA, are induced by *R. solanacearum* infection in pepper (Yang et al., 2021). In tomatoes, RSSC infection significantly enhances the expression of SA synthesis-related genes (*SlSARD1* and *SlPAL3*) and SA content (Wang et al., 2020; Li P. et al., 2025; Zhu et al., 2025). Other hormones such as abscisic acid (ABA) and ethylene are also associated with tomato resistance to *R. solanacearum* (Wang et al., 2025).

RNA-seq is widely used to analyse large-scale transcriptional regulation during tomato-RSSC interactions (Esposito et al., 2008; Ishihara et al., 2012; Takahashi et al., 2014; French et al., 2018; Chen et al., 2022), providing insights into defense mechanisms and the exploration of important genetic resources. However, relatively few transcriptomic studies have focused on bacterial wilt-resistant tomato cultivars. In this study, we found that disease-resistant cultivars “ZJ-7” and “04056” exhibited significantly higher resistance to bacterial wilt than AC. Subsequently, we identified the differentially expressed genes (DEGs) and the associated signaling and metabolic pathways in the disease-resistant cultivars “ZJ-7” and “04056” upon *R. solanacearum* infection. By comparing with the susceptible cultivar AC, we found that both disease-resistant cultivars were induced to express a greater number of resistance-related transcription factors, with earlier upregulation, more robust *PR* gene expression, and activation of the ethylene signalling pathway. These findings provide important theoretical support for breeding bacterial wilt-resistant tomato cultivars.

## Results

### Bacterial wilt resistance of tomato disease-resistant cultivars “ZJ-7” and “04056” is significantly higher than that of AC

**Figure 1:**
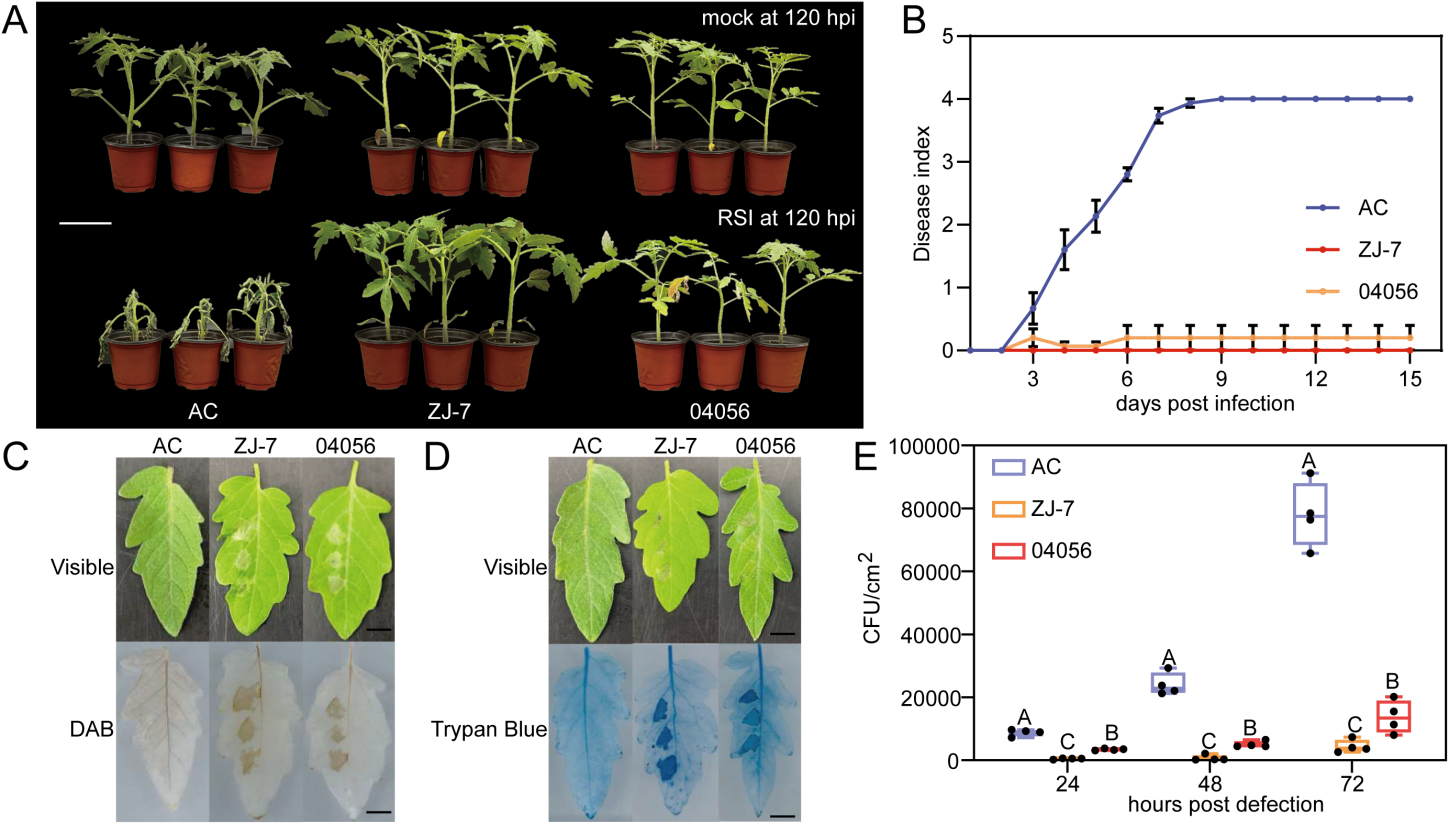
Bacterial wilt resistance and associated defence responses of “ZJ-7” and “04056” are superior to those of the susceptible tomato AC. **(A)** Phenotypic observations of tomato AC, “ZJ-7” and “04056” at 120 hpi with *R. solanacearum* strain FJ-91. Scale bar, 5 cm. **(B)** Disease indices of AC, “ZJ-7” and “04056” at 3 to 15 days post infection (dpi) with *R. solanacearum* FJ-91. Data were collected from 12 plants per genotype and shown as mean ± standard deviation (SD). **(C, D)**, Activation of HR in AC, “ZJ-7” and “04056” leaves following *R. solanacearum* FJ-91 infiltration. DAB staining to detect hydrogen peroxide (H_2_O_2_) accumulation and Trypan blue staining to visualize HR-related cell death were shown in C and D, respectively. Scale bar, 10 cm. **(E)** Quantification of bacterial growth (expressed as colony-forming units, CFU) in AC, “ZJ-7” and “04056” leaves at 24, 48 and 72 hpi with *R. solanacearum* FJ-91. Data are presented as mean ± standard error of four biological replicates. Different uppercase letters above the bars indicate significant differences determined by t-test (*p* < 0.01). *R. solanacearum* inoculation in panels **(A, B)** was performed by root irrigation, while infiltration in panels **(C–E)** was conducted via leaf injection, as described in Materials and Methods. hpi, hours post-infection. The disease index and phenotypes were derived from two independent experiments.

To evaluate the bacterial wilt resistance of disease-resistant cultivars “ZJ-7” and “04056” (generously provided by Zhejiang Academy of Agricultural Sciences), the susceptible tomato AC was used as a control. After 30 days of growth, the AC, “ZJ-7” and “04056” plants were inoculated with the *R. solanacearum* strain FJ-91 via root irrigation, followed by continuous phenotypic observation. By 120 hours post-inoculation (hpi), all AC plants had completely wilted and died, whereas “ZJ-7” and “04056” plants maintained healthy phenotypes (**Figure 1A**).

During the observation period, disease indices were calculated to quantify resistance levels. The results showed that “ZJ-7” and “04056” had significantly lower disease indices than AC. Notably, “ZJ-7” exhibited the strongest resistance with a disease index of 0 (**Figure 1B**). For “04056”, the observed fluctuation in its disease index can be attributed to mild wilting symptoms that appeared in a subset of plants at 3 hpi with *R. solanacearum*. These findings confirm that both “ZJ-7” and “04056” possess robust resistance to *R. solanacearum*.

To further validate the resistance mechanism, leaf injection assays were performed. The left side of the leaves from AC, “ZJ-7” and “04056” was inoculated with *R. solanacearum* strain FJ-91, while the right side was injected with sterile water as a negative control. Phenotypic analysis at 24 hpi showed distinct transparent plaques, a characteristic hallmark of hypersensitive response (HR), adjacent to the inoculation sites on “ZJ-7” and “04056” leaves. In contrast, no obvious HR-related plaques were observed on AC leaves (**Figure 1C**). Trypan blue and diaminobenzidine (DAB) staining were conducted to visualize defence responses. The results revealed that FJ-91 inoculation rapidly induce dense HR-like cell death and intense ROS burst in “ZJ-7” and “04056”, whereas these responses were significantly delayed and weaker in AC (**Figure 1D**). These data indicate that compared to the susceptible AC, *R. solanacearum* strain FJ-91 can trigger faster and stronger HR and ROS burst in the disease-resistant cultivars “ZJ-7” and “04056”.

To further confirm whether HR and ROS burst contribute to limiting the spread of *R. solanacearum*, colony-forming unit (CFU) counting was performed to quantify the growth of *R. solanacearum* in inoculated leaves. Consistent with the phenotypic and staining results, “ZJ-7” and “04056” exhibited significantly lower *R. solanacearum* growth and accumulation levels compared to AC. Since HR-like cell death and ROS burst are well-documented plant survival strategies that prevent pathogen invasion of distal tissues (Shi et al., 2023), these results further support that “ZJ-7” and “04056” achieve resistance to *R. solanacearum* FJ-91 by triggering HR and ROS burst, which effectively inhibit *R. solanacearum* proliferation and spread.

### Transcriptomic changes at the early stage of “ZJ-7” and “04056” in response to *R. solanacearum* infection

Activation of the plant immune system is typically accompanied by extensive changes in gene transcription, during which key disease-resistance genes are usually transcriptionally induced. To investigate the transcriptional regulation events occurring in tomato disease-resistant cultivars “ZJ-7” and “04056” upon *R. solanacearum* infection, we collected root samples at 3 and 6 hpi for transcriptome sequencing.

**Figure 2:**
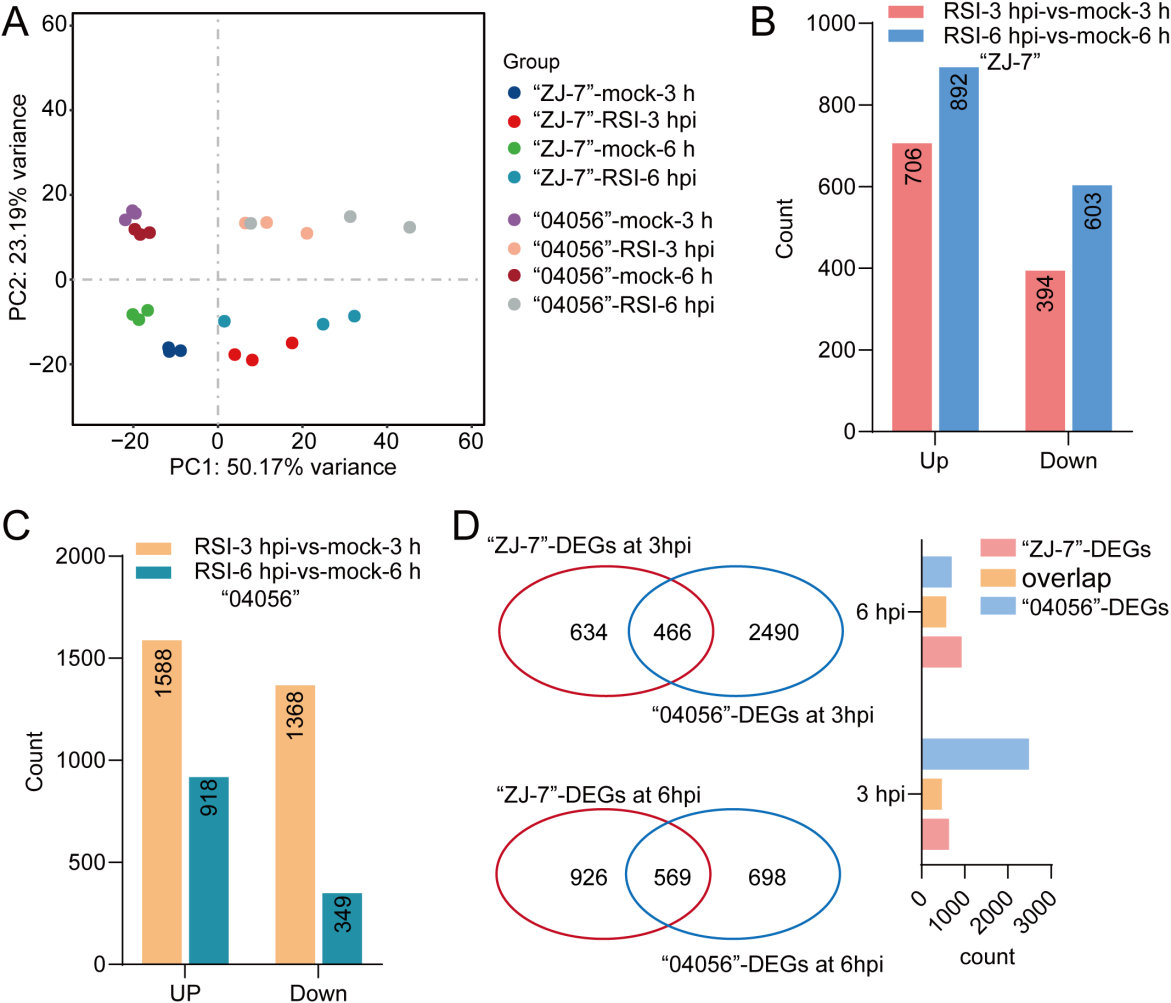
Transcriptomic profiling of “ZJ-7” and “04056” in response to *R. solanacearum* infection. **(A)** Principal component analysis (PCA) of RNA-seq data from roots collected at 3 and 6 hpi. Gene expression changes were investigated at 3 and 6 hpi. The analysis was performed using normalized expression level (FPKM) of all mapped genes. **(B)** Number of DEGs identified in “ZJ-7” at 3 and 6 hpi. **(C)** Number of DEGs identified in “04056”at 3 and 6 hpi. **(D)** Venn diagrams comparing DEG profiles between “ZJ-7” and “04056” at each time point. hpi, hours post-infection.

High-throughput sequencing of root cDNA was conducted using the Illumina Novaseq 6000 sequencing platform. After quality control of the raw data, which include the removal of adapter sequences and low-quality reads, a total of 162.32 Gb of clean reads were obtained (**Supplementary Table S1**). Each sample yielded at least 6.02 Gb, with a Q30 score exceeding 95.5% and GC content above 42.5%, indicating high transcriptome sequencing quality. Alignment of clean reads to the tomato reference genome (https://www.ncbi.nlm.nih.gov/datasets/genome/?taxon=4081) resulted in mapping rates ranging from 92.0% to 93.29%. Principal component analysis (PCA) was performed to assess global transcriptional changes and sample clustering among infected and control groups across time points. The first two principal components, PC1 and PC2, expalined 20.13% and 50.19% of the total transcriptional variance, respectively. The clear separation between groups and high intra-group clustering consistency reflected strong biological reproducibility (**Figure 2A**).

DEGs were identified using the thresholds of FDR < 0.05 and |log_2_ fold change (FC)| ≥ 1, with uninfected samples serving as controls (**Supplementary Table S4**). In “ZJ-7” roots, 1, 100 DEGs were identified at 3 hpi, including 706 upregulated and 394 downregulated genes. By 6 hpi, the number of DEGs increased to 1, 495, with 892 upregulated and 603 downregulated genes (**Figure 2B**). In “04056”, 2, 956 DEGs were identified at 3 hpi (1, 588 upregulated, 1, 368 downregulated), which increased to 1, 267 DEGs by 6 hpi (918 upregulated and 349 downregulated) (**Figure 2C**). Comparative analysis revealed 466 and 569 shared DEGs between the two cultivars at 3 and 6 hpi, respectively (**Figure 2D**). These common DEGs may contribute to the robust bacterial wilt resistance observed in both cultivars. Together, these results demonstrate that both “ZJ-7” and “04056” undergo extensive transcriptomic reprogramming in response to *R. solanacearum* infection, and the identified DEGs are likely invovled in mediating their early immune responses.

### GO functional enrichment analysis of differentially expressed genes in “ZJ-7” and “04056” under *R. solanacearum* inoculation

**Figure 3:**
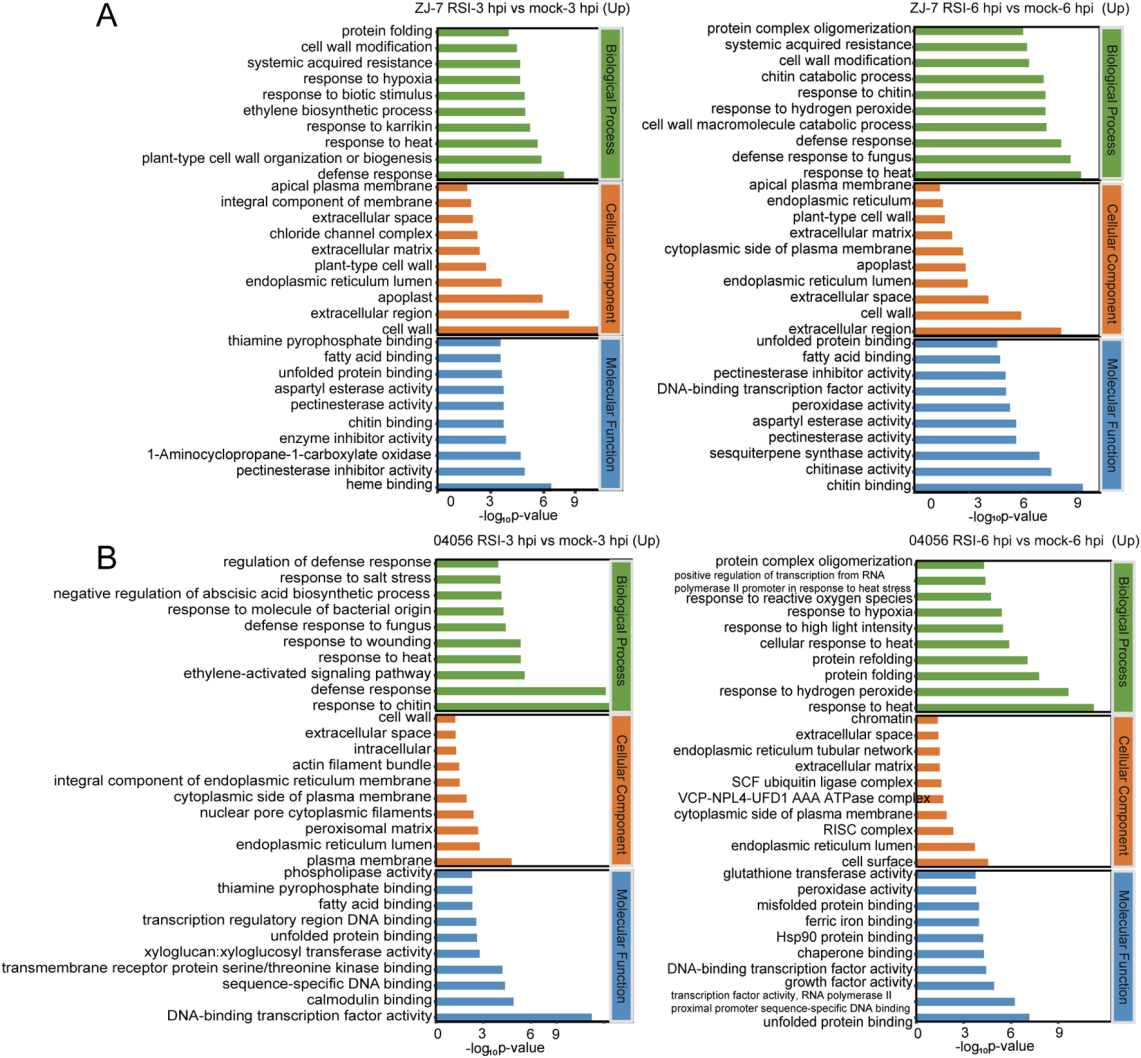
GO enrichment analysis of upregulated DEGs in “ZJ-7” and “04056” up-regulated DEGs identified at 3 and 6 hpi with *R. solanacearum*. **(A)** Top enriched GO terms of upregulated DEGs in “ZJ-7” at 3 hpi (left panel) and 6 hpi (right panel) post *R. solanacearum* inoulation. **(B)** Top enriched GO terms of upregulated DEGs in “04056” at 3 (left panel) and 6 (right panel) hpi post *R. solanacearum* inoculation. Mock, normal condition; RSI, *R. solanacearum* infection. hpi, hours post-infection.

To characterize the DEGs in “ZJ-7” and “04056” following *R. solanacearum* inoculation, we performed GO functional enrichment analysis (**Supplementary Table S5**). To clarify the affected biological processes (BP), cellular components (CC), and molecular functions (MF) in “ZJ-7” and “04056” post-inoculation, DEGs were analyzed at 3 and 6 hpi. The upregulated DEGs at 3 hpi in “ZJ-7” were significantly enriched in pathogen defense-related terms, including “systemic acquired resistance”, “response to biotic stimulus”, “defense response”, and “defense response to fungus” (**Figure 3A**). This indicates that the activation of “ZJ-7” rapidly activates its defense response against *R. solanacearum* at this early stage. Consistent with the results of “ZJ-7”, Among the top 30 enriched terms, upregulated DEGs of “04056” at 3 hpi were significantly enriched in defense-related terms, including “regulation of defense response”, “response to bacterial molecules”, “defense response”, and “fungal defense response” (**Figure 3B**). We also noticed that ROS-related terms (e.g., “response to hydrogen peroxide”) were enriched among the upregulated DEGs of “ZJ-7” and “04056”, aligning with the DAB staining results (**Figure 1D**). The significant enrichment in ROS-related terms suggest a strong correlation of “ZJ-7” and “04056” resistance with HR-induced ROS burst (**Figures 3A, B**).

Similar to “ZJ-7”, upregulated DEGs of “04056” at 3 hpi were also enriched in terms closely associated with transcription factors, including “sequence-specific DNA binding”, “DNA binding to transcription regulatory region”, and “DNA-binding transcription factor activity”. Notably, the enrichment of upregulated DEGs in TF-related terms persisted until 6 hpi (**Figure 3B**), indicating that rapid transcriptional activation of transcription factors involved in response to *R. solanacearum* infection may be crucial for bacterial wilt resistance in tomato.

Additionally, at both 3 and 6 hpi, the term “response to high temperature” and several organelle-related components, such as “cell wall”, “endoplasmic reticulum lumen”, and “apical plasma membrane”, were enriched among upregulated DEGs in “ZJ-7” and “04056” (**Figures 3A, B**). These results suggest that changes, restoration, and remodeling of cellular components may occur during the tomato response to *R. solanacearum* infection, which could help mitigate pathogen-induced cellular damage.

In “ZJ-7”, downregulated DEGs were significantly enriched in light-related terms, including “response to red light”, “photosystem II assembly”, “photoprotection”, “response to high light”, “photosynthesis”, and “light stimulus” (**Supplementary Figure S1A**). Additionally, at 3 hpi, downregulated DEGs in “ZJ-7” were enriched in terms such as “response to water deprivation”, “aquaporin activity”, “iron ion transmembrane transporter activity”, and “iron ion binding” (**Supplementary Figure S1B**). Compared with upregulated DEGs, downregulated DEGs showed no obvious commonalities between “ZJ-7” and “04056”. Unlike ZJ-7, no photosynthesis-related terms were enriched in downregulated DEGs of “04056” at 3 or 6 hpi; instead, terms closely related to plant growth and nutrition metabolism, such as “regulation of meristem growth”, “nitrate transport”, and “nitrate assimilation”, were enriched (**Supplementary Figure S1B**). Previous studies have demonstrated that nitrogen signaling is negatively correlated with tomato resistance to *R. solanacearum*; thus, “04056” may enhance its bacterial wilt resistance by suppressing nitrogen signaling.

In conclusion, despite significant differences in the enriched terms of downregulated DEGs between “ZJ-7” and “04056”, these downregulated DEGs are all closely associated with normal physiological metabolic activities. This reflects the trade-off between plant immune system activation and the maintenance of normal growth and development processes during plant immune system activation and the maintenance of normal growth and development processes during pathogen infection.

### KEGG pathway enrichment analysis of differentially expressed genes between “ZJ-7” and “04056” under *R. solanacearum* inoculation

**Figure 4:**
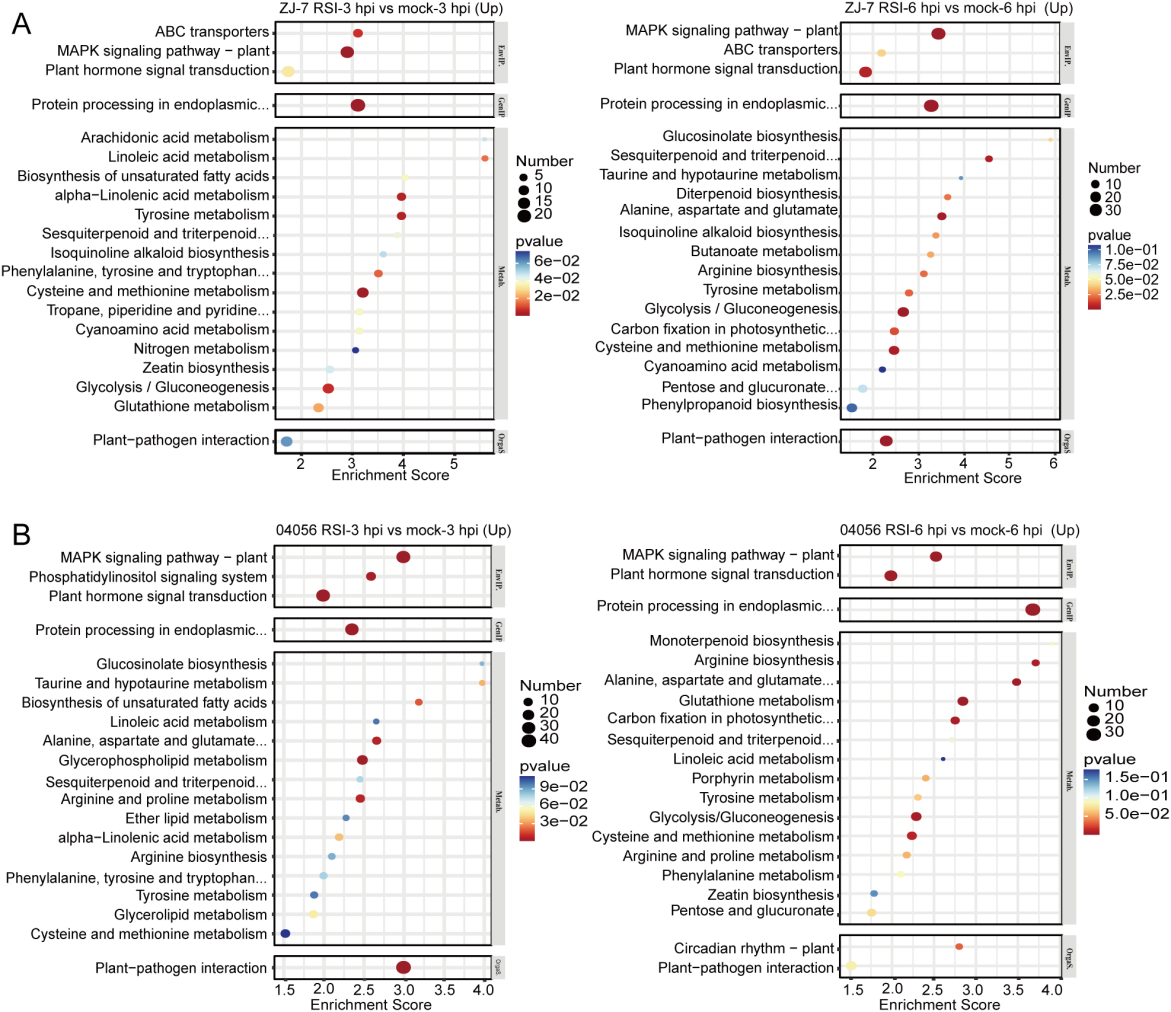
KEGG pathway enrichment analysis of up-regulated DEGs in”ZJ-7” and “04056” at 3 and 6 hpi following *R. solanacearum* infection. **(A)** Top KEGG pathways significantly enriched in up-regulated DEGs of “ZJ-7” at 3 hpi (left panel) and 6 hpi (right panel) with *R. solanacearum*. **(B)** Top KEGG pathways significantly enriched in up-regulated DEGs of “04056” at 3 hpi (left panel) and 6 hpi (right panel) with *R. solanacearum*. Mock, normal condition; RSI, *R. solanacearum* infection. hpi: hours post-inoculation.

To further clarify the signaling pathways mediating the response of tomato disease-resistant cultivars “ZJ-7” and “04056” to *R. solanacearum* infection, we performed KEGG signaling pathway enrichment analysis on the DEGs of the two cultivars at 3 and 6 hpi (**Supplementary Table S6**). The top 20 most significantly enriched KEGG pathways for upregulated DEGs in “ZJ-7” were presented in **Figure 4**. Consistent with the GO enrichment results, KEGG analysis revealed that upregulated DEGs in “ZJ-7” were significantly enriched in two core plant disease resistance-related signaling pathways, including “Plant MAPK signaling pathway” and “Plant-pathogen interaction”, at both 3 and 6 hpi (**Figure 4A**). Consistent with “ZJ-7”, the upregulated DEGs in “04056” were significantly enriched in the “plant MAPK signaling pathway” and “plant-pathogen interaction” pathway at both 3 and 6 hpi with *R. solanacearum* (**Figure 4B**). This indicates that *R. solanacearum* infection uniformly activates the expression of defense-related genes across different disease-resistant tomato cultivars.

Beyond these direct immune-related signaling pathways, several amino acid metabolism pathways were also enriched among the upregulated DEGs in “ZJ-7” at both time points, including “Tyrosine metabolism”, “Phenylalanine, tyrosine and tryptophan biosynthesis”, “Alanine, aspartate and glutamate metabolism”, “Arginine biosynthesis”, and “Cysteine and methionine metabolism” (**Figure 4A**). Similarly, consistent with the results of “ZJ-7”, amino acid metabolic pathways were significantly enriched among the upregulated DEGs in “04056”, with a high degree of overlap with those in “ZJ-7”, including “tyrosine metabolism”, “phenylalanine, tyrosine and tryptophan biosynthesis, alanine, aspartate and glutamate metabolism”, “arginine biosynthesis”, and cysteine and “methionine metabolism” (**Figure 4B**). This highlights a universal association between these amino acid metabolic pathways and the establishment of tomato bacterial wilt resistance. These amino acids can act as critical precursors for basal defense responses in tomato against *R. solanacearum*. For instance, key components of the tyrosine metabolism pathway are induced by JA and closely linked to plant disease resistance.

Phytoalexins, typically synthesized upon pathogen perception, are pivotal secondary metabolites for plant defense against pathogen infection. Current studies indicate that phytoalexin synthesis is tightly regulated by ethylene and JA signals, with close ties to terpenoid metabolism. At 6 hpi with *R. solanacearum*, the pathways “Sesquiterpenoid and triterpenoid biosynthesis” and “Diterpenoid biosynthesis” were significantly enriched in “ZJ-7”. Given their intimate association with phytoalexin synthesis, these results suggest that “ZJ-7” had initiated phytoalexin production to counter *R. solanacearum* infection at this stage (**Figure 4A**). Notably, the “sesquiterpenoid and triterpenoid biosynthesis” pathway was activated in “04056” at 3 hpi, earlier than that in “ZJ-7”. Furthermore, both “sesquiterpenoid and triterpenoid biosynthesis” and “monoterpenoid biosynthesis” pathways were significantly enriched in “04056” at 6 hpi (**Figure 4B**). This implies that “04056” can initiate phytoalexin synthesis more rapidly and sustain this process more effectively compared to “ZJ-7”.

Collectively, these results demonstrate that the transcriptionally activated DEGs in both “ZJ-7” and “04056” upon *R. solanacearum* infection are primarily involved in relatively similar signaling pathways.

KEGG enrichment analysis of the downregulated DEGs of “ZJ-7” showed conistent results with GO enrichment analysis: photosynthesis-related terms were significantly enriched, alongside inhibited biosynthesis of antenna proteins and carotenoids (**Supplementary Figure S2A**). At 6 hpi, additional secondary metabolic pathways were suppressed, including “cutin, suberin, and wax biosynthesis”, “tropane, piperidine, and pyridine alkaloid biosynthesis”, and “porphyrin metabolism”. This indicates that tomato undergoes substantial reprogramming of secondary metabolic pathways to prioritize phytoalexin synthesis, with the production of other metabolites being attenuated or halted.

Consistent with the GO enrichment findings, KEGG analysis also revealed substantial differences in downregulated DEGs between the two tomato cultivars. No significant enrichment of photosynthesis-related signaling pathways was observed in the downregulated DEGs of “04056” at 3 hpi or 6 hpi (**Supplementary Figure S2B**). Instead, amino acid metabolic pathways were highly enriched in the downregulated DEGs of “04056” post-infection, including “valine, leucine, and isoleucine biosynthesis”, “tryptophan metabolism”, “β-alanine metabolism”, “histidine metabolism”, “valine, leucine, and isoleucine degradation”, “arginine and proline metabolism”, “phenylalanine metabolism”, and “cysteine and methionine metabolism”. This suggests that amino acid metabolic processes may exert a cultivar-specific regulatory role in the resistance mechanism of “04056” (**Supplementary Figure S2B**).

### Enhanced stablilty of *PR* gene expression in “ZJ-7” and “04056” under *R. solanacearum* inoculation

**Figure 5:**
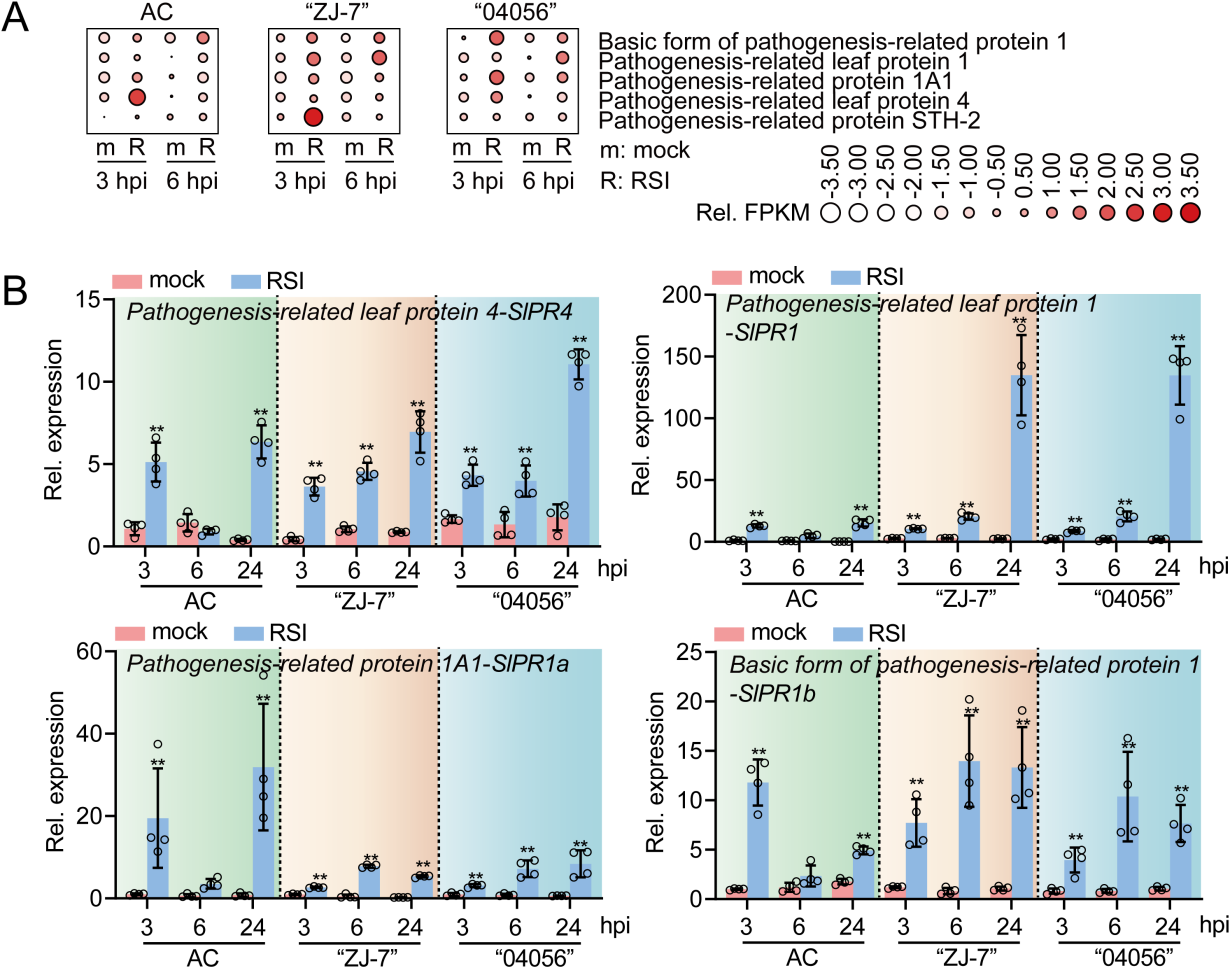
Expression analysis of *PR* genes in AC, “ZJ-7” and “04056” at 3 and 6 hpi with *R. solanacearum*. **(A)** Relative expression of the identified *PR* genes in AC, “ZJ-7” and “04056” revealed by heatmap analysis. The relative expression levels were normalized based on FPKM obtained by RNA-seq. **(B)** Relative expression of the selected *PR* genes analyzed by RT-qPCR. Data presented are mean standard error of four replicates. Asterisk above the bars indicate significant differences among means (*p* < 0.01) by t-test. Mock, normal growth condition; RSI, *R. solanacearum* infection. hpi: hours post- infection.

As previously indicated, GO enrichment analysis of up-regulated DEGs in the roots of disease-resistant cultivars “ZJ-7” and “04056” at 3 and 6 hpi with *R. solanacearum* revealed significant overrepresentation of terms associated with plant disease resistance responses, pathogen responses, and transcriptional regulation. Consistently, KEGG pathway analysis highlighted significant enrichment in the “Plant MAPK signaling pathway” and “Plant-pathogen interaction” pathways. Examination of the genes constituting these enriched categories identified a predominance of *PR* genes and transcription factors among the up-regulated DEGs. Given that *PR* genes are pivotal executers of plant disease resistance, we compared their expression patterns, obtained from our current transcriptome data, with those from our previously generated root transcriptome of the susceptible cultivar AC under identical infection conditions. Notably, at 3 hpi, the expression of these PR genes was significantly upregulated in AC, to a similar extent as in the disease-resistant cultivars “ZJ-7” and “04056” (**Figure 5A**). This suggests that the intial induction of these PR genes is common early response and is not the primary determinant conferring differential resistance against bacterial wilt. However, a key distinction emerged at 6 hpi: the elevated expression of these PR genes was sustained in the disease-resistant cultivars “ZJ-7” and “04056”, but not in AC, where it markedly declined. This indicates a more stable and prolonged activation of the PR gene-mediated defense response in the disease-resistant cultivars.

This speculation was corroborated by RT-qPCR analysis of selected PR genes *SlPR4*, *SlPR1*, *SlPR1a*, and *SlPR1b*. In lines ZJ-7” and “04056”, these genes maintained high expression levels at at 3, 6, and 24 hpi with *R. solanacearum*. In contrast, their transcriptional activation in AC was absent or not sustained by 6 hpi (**Figure 5B**). We therefore propose that the failure of the susceptible cultivar AC to maintain *PR* gene expression beyond the initial infection phase may preclude the establishment of a stable and effective resistance, thereby facilitating successful infection by *R. solanacearum*.

### Abundant upregulation of transcription factors may confer more robust PR gene expression in “ZJ-7” and “04056”

**Figure 6:**
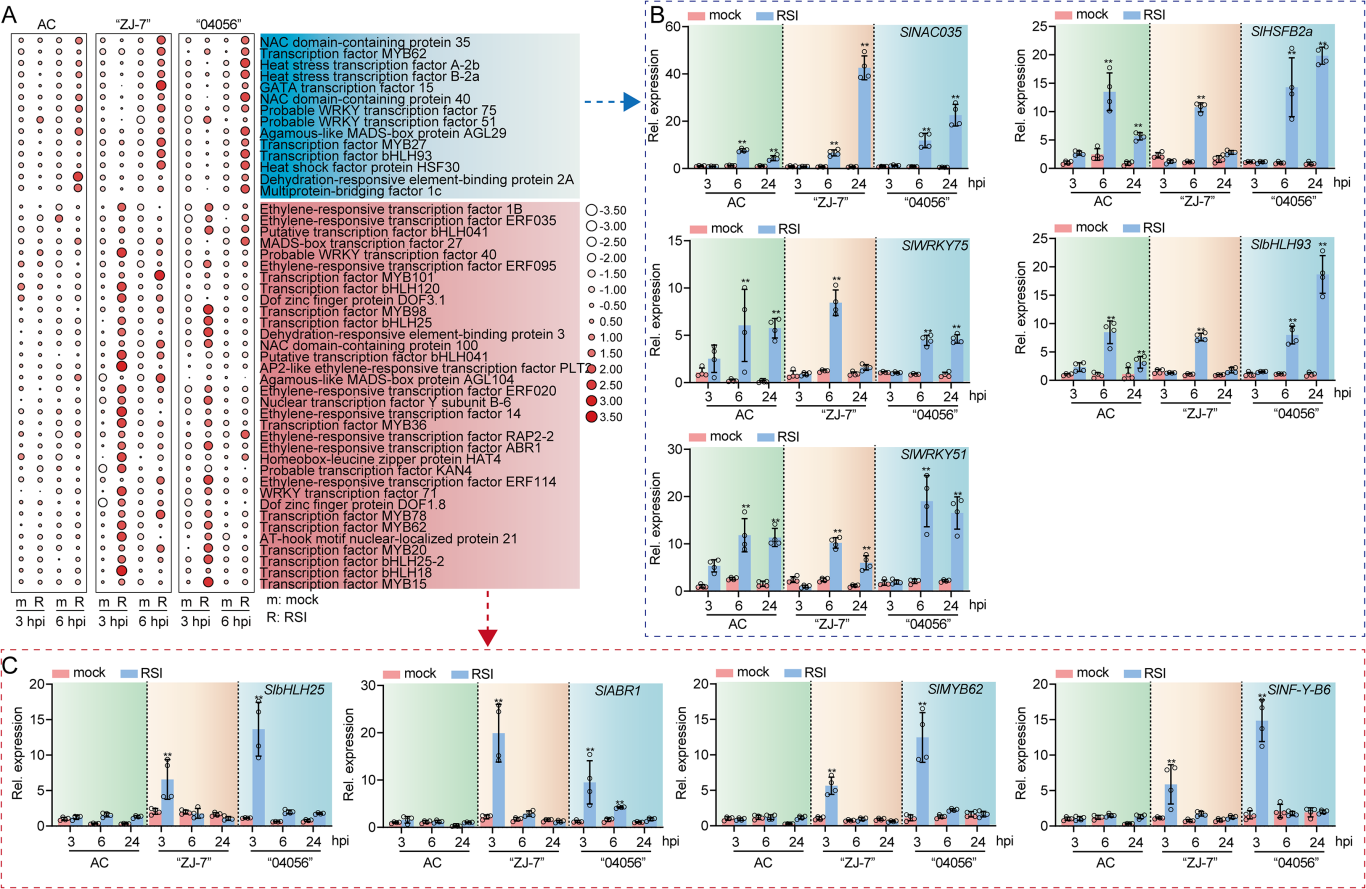
Expression analysis of transcription factors in AC, “ZJ-7” and “04056” at 3 and 6 hpi with *R. solanacearum*. **(A)** Relative expression of the genes encoding transcription factors in AC, “ZJ-7” and “04056” revealed by heatmap analysis. The relative expression levels were normalized based on FPKM obtained by RNA-seq. **(B)** RT-qPCR validation of transcription factors up-regulated at 6 hpi across all genotypes. **(C)** RT-qPCR validation of transcription factors specifically up-regulated at 3 hpi in the disease-resistant cultivars “ZJ-7” and “04056”. Relative expression of the selected genes encoding transcription factors analyzed by RT-qPCR. Expression patterns for each gene set are summarized in the adjacent blue **(B)** and red **(C)** panels, respectively. Data in **(B, C)**, are presented as mean ± standard error of four replicates. Asterisk above the bars indicate significant differences between mock and infected conditions within each cultivar at the same time point (*p < 0.01). Mock, normal growth condition; RSI, *R. solanacearum* infection. hpi: hours post- infection.

The expression of PR genes is known to be precisely regulated by transcription factors (Ngou et al., 2021). In addition to *PR* genes, we observed substantial upregulation of numerous transcription factors in the disease-resistant cultivars “ZJ-7” and “04056”. Further analysis of TF expression patterns across AC, “ZJ-7”, and “04056” revealed marked differences (**Figure 6A**). In the susceptible cultivar AC, only a limited set of transcription factors was induced upon *R. solanacearum* infection, and this induction was delayed until 6 hpi in all three genotypes. In contrast, the disease-resistant cultivars “ZJ-7” and “04056” exhibited a distinct quantitative and temporal advantage, with a broader suite of transcription factors being activated specifically at 3 hpi.

We selected five transcription factors, *SlNAC035*, *SlHSFB2a*, *SlWRKY75*, *SlbHLH93*, and *SlWRKY51*, that were upregulated at 6 hpi in all genotypes and validated their expression by qRT-PCR in ‘AC’, “ZJ-7”, and “04056” at 3, 6, and 24 hpi (**Figure 6B**). Their expression dynamics were consistent with transcriptomic data, showing sustained upregulation through 24 hpi.

Additionally, we analyzed four transcription factors, *SlbHLH25*, *SlABR1*, *SlMYB62*, and *SlNF-Y-B6*, that were specifically upregulated in “ZJ-7” and “04056” at 3 hpi (**Figure 6C**). RT-qPCR confirmed that these transcription factors were transcriptionally activated exclusively in the disease-resistant cultivars “ZJ-7” and “04056” at 3 hpi, with no comparable induction observed in AC. We hypothesize that this early and specific activation of transcription factors contributes to the stable and enhanced expression of *PR* genes in “ZJ-7” and “04056” at later stages, thereby reinforcing their defense response against bacterial wilt.

### Ethylene signaling is activated in “ZJ-7” and “04056” upon *R. solanacearum* infection

In addition to differential expression of the genes encoding PR proteins and transcription factors, we observed that functional terms and pathways associated with ethylene biosynthesis were significantly activated in the disease-resistant cultivars “ZJ-7” and “04056” at 3 hpi with *R. solanacearum*. This finding suggests that ethylene biosynthesis may play a crucial role in mediating tomato resistance to bacterial wilt.

**Figure 7:**
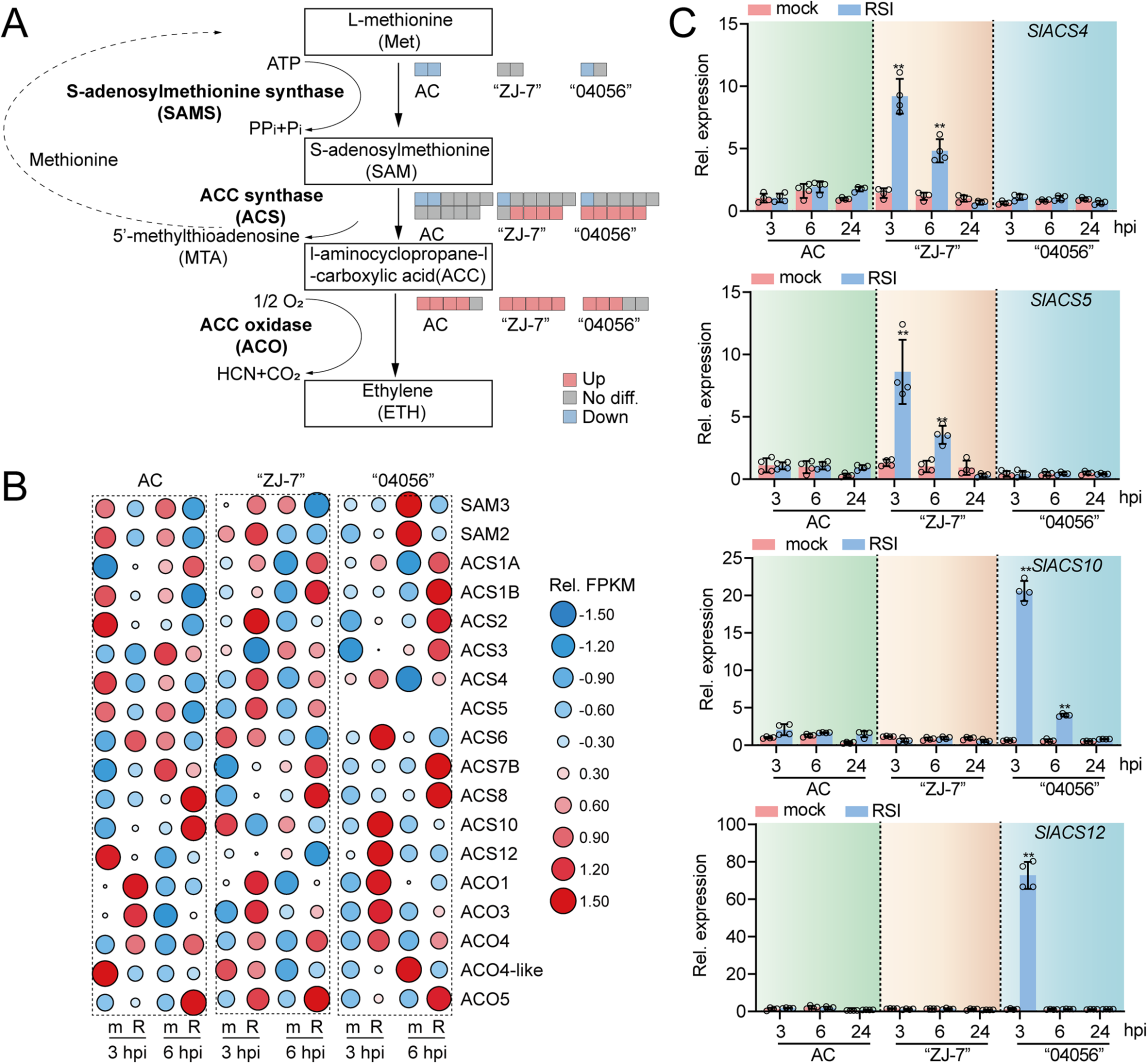
Expression analysis of ethylene biosynthesis pathway-related genes in AC, “ZJ-7” and “04056” at 3 and 6 hpi with *R. solanacearum*. **(A)** The expression pattern of DEGs involved in ethylene biosynthesis in AC, “ZJ-7” and “04056”. Red, blue, and gray boxes depict the up-regulated, down-regulated, and non-differentially expressed DEGs. **(B)** Heatmap showing relative expression of the ethylene biosynthesis pathway-related genes in AC, “ZJ-7” and “04056”. The relative expression levels were normalized based on FPKM obtained by RNA-seq. M, normal growth condition; R, *R. solanacearum* infection. **(C)** Relative expression of ACS-encoding genes determined by RT-qPCR. Gene expression patterns are summarized in the red panel. Data are presented as the mean ± standard error of four replicates. Asterisk above the bars indicate significant differences determined by t-test (*p* < 0.01). Mock, normal growth condition; RSI, *R. solanacearum* infection. hpi: hours post- infection.

To exlore this hypothesis, we analyzed the expression of ethylene biosynthesis pathway-related genes in the susceptible cultivar AC and the disease-resistant cultivars “ZJ-7” and “04056” during the early stage of *R. solanacearum* infection. The ethylene biosynthesis pathway involves three key enzymes: S-adenosylmethionine synthase (SAMS), 1-aminocyclopropane-1-carboxylic acid (ACC) synthase (ACS), and ACC oxidase (ACO) (Larsen, 2015). We first identified homologous genes of these three enzymes in the tomato genome and analyzed their FPKM values in tomato roots at 3 and 6 hpi with *R. solanacearum*, aiming to clarify which ethylene biosynthesis genes are transcriptionally activated during the early infection stage. Regarding two *SAMS* genes, their expression exhibited a clear down-regulation trend in AC, but showed inconsistent expression patterns in “ZJ-7” and “04056”. Notably, while both genes demonstrated significant upregulation in “ZJ-7” at 3 hpi, their expression was downregulated in “04056” at 6 hpi with *R. solanacearum* (**Figures7A, B**). These results indicate that *R. solanacearum* infection leads to stable transcriptional downregulation of two *SAMS* genes in AC. In contrast, the expression of *ACS* genes was significantly affected by *R. solanacearum* infection. In the susceptible cultivar AC, two *ACS* genes was transcriptionally inhibited by *R. solanacearum* infection. By comparison, in the disease-resistant cultivars, only one *ACS* gene was transcriptionally repressed in “ZJ-7”, while the other 4 *ACS* genes were significantly upregulated in both “ZJ-7” and “04056” (**Figure 7A**). Notably, the specific *ACS* genes responsive to *R. solanacearum* differed between the two resistant cultivars. In “ZJ-7”, the expression levels of *SlACS1a*, *SlACS2*, *SlACS4* and *SlACS5* were significantly induced at 3 hpi. In “04056”, *SlACS1*, *SlACS3*, *SlACS6*, *SlACS10* and *SlACS12* were more strongly induced by the infection. These findings imply that although both resistant cultivars mount an ethylene-related defensive response, the distinct *ACS* gene subsets activated suggest they may employ divergent resistance mechanisms (**Figures 7A, B**).

As the final key enzyme that converts ACC to ethylene, ACO exhibited significantly upregulated expression in both disease-resistant and susceptible cultivars upon *R. solanacearum* infection. Thus, the differential expression of *ACS* genes among tomato cultivars may serve as the key determinant of whether ethylene synthesis is activated during the early stage of *R. solanacearum* infection.

To verify the transcriptome results, we performed RT-qPCR to analyze the expression of *ACS* genes that were strongly induced by *R. solanacearum*: *SlACS4* and *SlACS5* in “ZJ-7”, and *SlACS10* and *SlACS12* in “04056”. The results confirmed that the transcriptional activation of these *ACS* genes was restricted to the early infection stage, and the specifically activated *ACS* homologs differed significantly between “ZJ-7” and “04056”. Importantly, the qRT-PCR results were generally consistent with the transcriptome data (**Figure 7C**), validating the reliability of our initial findings.

**Figure 8:**
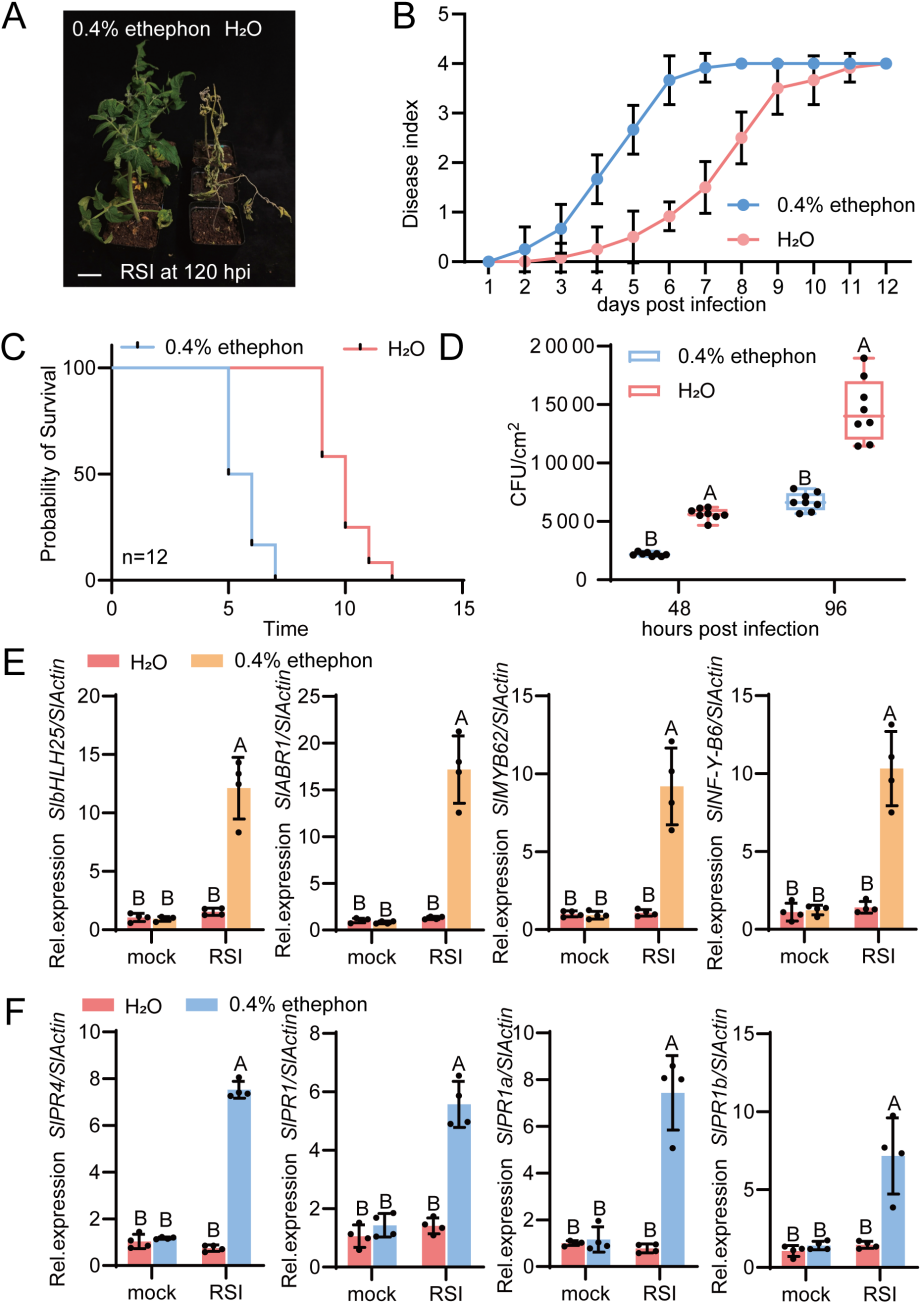
The role of ethylene in tomato defense against *R. solanacearum* infection. **(A)** Phenotypes of AC tomato plants treated with ethephon (ddH_2_O as mock control) under *R. solanacearum* infection **(RSI)** at 120 hpi. Scale bar, 5 cm. **(B)** Disease index of ethephon-treated and mock-treated (ddH_2_O) AC tomato plants. For RSI group, disease indices were calculated from 0 to 12 days post inoculation (dpi), with 12 plants per treatment dynamically scored throughout the period. **(C)** Survival rates of ethephon-treated and mock-treated (ddH_2_O) AC tomato plants during 12-days treatment of *R. solanacearum* infection. **(D)** Colonization levels of *R. solanacearum* in ethephon-treated and mock-treated (ddH_2_O) AC tomato plants, represented as cfu (clony-forming units) at 48 and 96 hours post inoculation. **(E)** Relative transcript levels of transcription factor genes (*SlbHLH25*, *SlABR1*, *SlMYB62*, and *SlNF-Y-B6*) in ethephon-treated AC plants at 3 hpi with *R. solanacearum*. **(F)** Relative transcript levels of *PR* genes (*SlPR4*, *SlPR1*, *SlPR1a* and *SlPR1b*) in ethephon-treated and mock-treated (ddH_2_O) AC tomato plants at 6 hpi with *R. solanacearum*. In **(E, F)**, data are presented as mean ± standard error of four biological replicates. Different uppercase letters above the bars indicate significant differences determined by t-test (p<0.01). Mock, normal growth condition; RSI, *R. solanacearum* infection. hpi: hours post- infection. The disease index and phenotypes were derived from two independent experiments.

To further verify the role of ethylene in mediating tomato resistance against bacterial wilt, we conducted a foliar spray treatment with ethephon (an ethylene-releasing compound) on the susceptible cultivar AC, followed by inoculation with *R. solanacearum*. Results showed that ethephon application significantly enhanced the resistance of AC to bacterial wilt (**Figure 8A**), improved its survival rate under *R. solanacearum* infection, and delayed the progression of disease symptoms (**Figures 8B, C**). Additionally, the colonization level of *R. solanacearum* in ethephon-treated AC plants was significantly lower than that in the untreated control group (**Figure 8D**).

To investigate the molecular mechanisms of ethephon-induced resistance, we quantified the expression of several *R. solanacearum*-responsive genes (**Figures 8E, F**), which were previously identified in the disease-resistant cultivars “ZJ-7” and “04056” using our RNA-seq data. We first tested the expression of four transcription factor genes, *SlbHLH25*, *SlABR1*, *SlMYB62*, and *SlNF-Y-B6*, in ethephon-sprayed AC at 3 hpi. The results showed that ethephon application significantly upregulated the transcriptional levels of these four genes in AC at 3 hpi (**Figure 8E**). We further examined the expression patterns of *PR genes*, including *SlPR4*, *SlPR1*, *SlPR1a*, and *SlPR1b*, in ethephon-sprayed AC at 6 hpi. It was found that the expression of these *PR* genes was also significantly enhanced in AC at 6 hpi with *R. solanacearum* inoculation (**Figure 8F**).

Collectively, these physiological and molecular evidence confirm that exogenous ethylene application can effectively improve the resistance of the susceptible cultivar AC to bacterial wilt. This further indicates that the deficiency in ethylene biosynthesis is a key factor contributing to the susceptibility of AC to *R. solanacearum*-induced bacterial wilt.

## Discussion

Bacterial wilt caused by *R. solanacearum* poses a severe threat to global tomato production, making the exploration of resistance mechanisms and susceptible determinants crucial for disease management. In this study, we employed transcriptome sequencing to systematically dissect the transcriptional regulatory networks underlying bacterial wilt resistance in two disease-resistant tomato (“ZJ-7” and “04056”) and to clarify the resistance-related transcriptional characteristics of them. Our analyses identified two key insights into tomato-*R. solanacearum* interactions: first, the success of resistant tomato (“ZJ-7” and “04056”) to sustain immune responses is closely associated with the sufficient expression of specific transcription factors, which in turn improves the transcriptional activation of *PR* genes; second, ethylene signaling plays an indispensable regulatory role in mediating the resistance of “ZJ-7” and “04056” against *R. solanacearum* infection. These findings collectively provide novel molecular perspectives on tomato bacterial wilt resistance and susceptibility.

### Consistency in upregulated DEGs underlies bacterial wilt resistance in “ZJ-7” and “04056”

Pathogen invasion induces extensive changes in plant cellular gene expression levels, which are crucial to the establishment of disease resistance (Birkenbihl et al., 2017). We analyzed the root transcriptomes of two tomato disease-resistant cultivars (“ZJ-7” and “04056”) under both *R. solanacearum* inoculation and non-inoculated conditions. Both cultivars exhibited significant alterations in root gene expression at 3 and 6 hpi with *R. solanacearum*, consistent with observations in other Solanaceous crops, such as eggplant and pepper, upon *R. solanacearum* infection. For instance, 2896 DEGs were identified in eggplant roots post-infection, while in pepper cultivar CM334, 1400, 3335, 2878, and 4484 DEGs were detected in roots at 1, 3, 5, and 7 days post-inoculation (dpi), respectively (Du et al., 2021; Xiao et al., 2023). In our study, “ZJ-7” showed 1100 and 1485 DEGs at 3 and 6 hpi, respectively, while “04056” exhibited 2956 and 1267 DEGs at the same time points (**Figures 2B, C**). Similarly, in resistant potato cultivars CG and CR, 4011 and 2766 DEGs, respectively, were identified following *R. solanacearum* inoculation (Jose et al., 2023). These results indicate that the number of DEGs varies substantially among different resistant cultivars upon *R. solanacearum* inoculation, suggesting no direct correlation between DEG count and the development of bacterial wilt resistance.

Notably, GO enrichment analysis revealed high degree of consistency in the composition of upregulated DEGs between “ZJ-7” and “04056”, whereas significant differences were observed in downregulated DEG subsets. This finding aligns with previous studies on potato (Jose et al., 2023). In “ZJ-7” and the potato cultivar CG, downregulated DEGs were significantly enriched in terms related to light response, photosynthesis, and oxidative stress, aphenomenon not observed in “04056” and CR, another bacterial wilt-resistant potato cultivar (**Figures 3C, D**). Thus, similar to DEG count, the composition of downregulated DEGs does not appear to be a key determinant of bacterial wilt resistance in tomato and potato.

In contrast, the upregulated DEGs of “ZJ-7” and “04056” under *R. solanacearum* infection exhibited striking consistency, with co-enrichment in functional terms such as “regulation of defense response” and “response to bacterial molecules” (**Figures 3A, B**). In potatoes, the upregulated DEGs in *R. solanacearum* -resistant cultivars CG and CR were also enriched in terms related to “cell wall” (Jose et al., 2023), indicating that pathogen-induced upregulation of resistance-related genes is directly associated with plant disease resistance. Consistent with this, numerous genes closely linked to plant disease resistance, such as *CaHSFB2a* and *SlWRKY30*, show significant expression induction upon pathogen infection (Ashraf et al., 2018; Dang et al., 2023). This further supports the notion that transcriptional activation of disease resistance-related genes is a core step in the initiation of the plant immune system, and the consistent upregulation of these genes in “ZJ-7” and “04056” likely contributes to their shared resistance phenotype.

### Stable expression of PR genes: a key feature of resistance in “ZJ-7” and “04056”

PR proteins are a well-characterized class of immune-related molecules that play pivotal roles in initiating and amplifying plant immune responses. Under normal growth conditions, *PR* gene typically maintain low basal expression levels; however, upon perception of PAMPs or effector molecules, they are rapidly transcriptionally activated. The PR proteins encoded by these genes then exert direct or indirect antimicrobial activities, thereby enhancing plant disease resistance.

Our transcriptome data revealed a striking difference in PR gene expression dynamics between resistant and susceptible tomato cultivars. In “ZJ-7” and “04056”, PR genes remained stably transcriptionally activated at both 3 and 6 hpi with *R. solanacearum* (**Figures 5A, B**). In contrast, the same PR genes in the susceptible cultivar AC were only transiently activated at 3 hpi and failed to sustain expression at 6 hpi (**Figure 5B**). This observation aligns with previous studies linking PR gene expression to disease resistance across multiple plant species. For example, the expression levels of wheat T*aPR1*, *TaPR2*, and *TaPR5* are tightly correlated with its resistance to stripe rust (Qian et al., 2025), with resistant wheat cultivars consistently exhibiting higher *PR* gene expression than susceptible counterparts (Geng et al., 2022). Similarly, *PnPR4* confers broad-spectrum resistance to root rot in *Panax notoginseng* by mediating defense responses against soil-borne pathogens (Sun et al., 2024). These findings, coupled with our own, suggest that not only the the magnitude of *PR* gene expression but also the stability of their expression across different stages of pathogen infection is is a critical determinant of effective plant disease resistance.

The transcriptional activation of PR genes is mainly regulated by transcription factors. Upon pathogen infection, these transcription factors perceive upstream signals, undergo activation, and subsequently bind to the specific cis-acting elements in the of PR gene promoters. For instance, in apple, MdTGA2c-2 interacts with MdNPR1 under the induction of SA, forming a transcriptional complex that activates *MdPR1* expression and enhances resistance to powdery mildew invasion (Lan et al., 2025). During tomato-*Botrytis cinerea* interactions, SlERF.F4 directly binds to the promoter *SlPR-STH2* to drive its transcriptional activation, thereby positively regulating disease resistance (Li S. et al., 2025). Beyond direct regulation, transcription factors can also modulate *PR* gene expression through indirect pathways. For instance, SlWRKY71 activates *SlDCD1* expression to promote endogenous H_2_S production. H_2_S then act as a signaling molecule to induce *SlPR1* expression, ultimately enhancing tomato resistance to *Pseudomonas syringae* pv. tomato (Pst.) DC3000 (Zhao et al., 2025). This indicates that he diverse and extensive regulatory networks through which transcription factors control PR gene expression.

Our analysis revealed that, compared with the susceptible AC, the resistant cultivars “ZJ-7” and “04056” exhibited significantly higher expression of numerous transcription factor genes in roots at 3 hpi with *R. solanacearum* (**Figure 6**). Among these, transcription factors such as bHLH25 and ABR1 have been previously implicated in plant immune responses (Choi and Hwang, 2011; Bi and Zhou, 2025; Liao et al., 2025). Their early activation at 3 hpi may contribute to the sustained transcriptional activation of *PR* genes in “ZJ-7” and “04056” at 6 hpi. Additionally, a distinct subset of transcription factors was transcriptionally activated at 6 hpi; among these, factors like HSFB2a and WRKY51 have been confirmed to participate in plant defense responses against pathogens (Ashraf et al., 2018; Yang X. et al., 2023). Notably, these late-activated transcription factors were expressed in AC, “ZJ-7”, and “04056”, suggesting they may mediate conserved immune processes in tomato following *R. solanacearum* infection after 6 hpi.

This stage-specific activation of transcription factors, with early induction in resistant cultivars driving sustained PR gene expression, supports the notion that plant immune responses are characterized by both temporal specificity and functional continuity (Birkenbihl et al., 2017; Jones et al., 2024). The failure of AC to maintain transcription factor expression at 6 hpi likely contributes to its inability to sustain *PR* gene activation, thereby rendering it susceptible to *R. solanacearum* infection.

### Ethylene signaling regulates PR gene expression via activating transcription factors

Beyond defense-related genes and transcription factors, our GO enrichment analysis revealed that ethylene signaling-related terms were significantly enriched among the upregulated DEGs in both “ZJ-7” and “04056” at 3 hpi with *R. solanacearum*. This finding points to a potential regulatory link between the ethylene signaling pathway and tomato bacterial wilt resistance, which we further validated by analyzing the expression of key ethylene biosynthesis genes. Our results showed that ACO, the final enzyme in ethylene biosynthesis, exhibited increased expression in both susceptible cultivar AC and the disease-resistant cultivars “ZJ-7” and “04056” at 3 hpi with *R. solanacearum*. In contrast, ACS, a rate-limiting enzyme upstream of ACO, was significantly upregulated only in “ZJ-7” and “04056”, but not in AC. Additionally, the expression level of SAMS was downregulated in AC roots under *R. solanacearum* infection (**Figure 7**). These expression patterns collectively suggest that AC fails to synthesize ethylene and activate downstream signaling pathways at 3 hpi, which may be a critical susceptibility determinant.

Ethylene is well-documented for its roles in plant growth and development (Dubois et al., 2018), and recent studies have increasingly highlighted its function as an early warning molecule and key signaling mediator in plant disease resistance (Binder, 2020; Ding et al., 2022; Waadt et al., 2022). A core mechanism by which ethylene enhances disease resistance is the induction of PR protein expression and accumulation, an effect that likely contributes to the sustained transcriptional activation of PR genes in “ZJ-7” and “04056”

To experimentally confirm the role of ethylene in tomato bacterial wilt resistance, we performed exogenous application of ethephon on the susceptible cultivar AC prior to R. solanacearum inoculation. Ethephon treatment significantly enhanced the resistance of AC to bacterial wilt, which was accompanied by two key molecular changes. First, the transcription factors *SlbHLH25*, *SlABR1*, *SlMYB62*, and *SlNF-Y-B6*, which are specifically upregulated in “ZJ-7” and “04056”, were transcriptionally activated at 3 hpi with *R. solanacearum* infection. Second, the transcriptional expression of PR genes, which is lost in AC at 6 hpi, was fully restored by ethephon treatment (**Figure 8**). These results directly demonstrate that ethylene signaling bridges the gap between transcription factor activation and sustained PR gene expression in resistant tomato cultivars.

Ethylene signaling does not act in isolation but rather crosstalks with multiple plant hormone signaling pathways to modulate defense responses (Li et al., 2019). For instance, during Xanthomonas spp. infection of cassava, enhanced endogenous ethylene synthesis activates the ERF transcription factor EIL5 (Wei et al., 2022; Zheng et al., 2024). EIL5 then interacts with the heat shock factor HSF30 to induce melatonin biosynthesis, which in turn promotes the expression of PR proteins. In Arabidopsis, ethylene and JA signaling synergistically induce the synthesis of the phytoalexin camalexin. The process involves the phosphorylation of the ERF transcription factor ERF1 by mitogen-activated protein kinases MPK3/MPK6, which then interacts with WRKY33 to activate camalexin biosynthesis genes (Zhou et al., 2022). In tomatoes, overexpression of the ERF transcription factor SlERFA3 significantly enhances bacterial wilt resistance by altering root exudates and modulating the soil microbial community, an effect tightly linked to ethylene signaling (Yang K. et al., 2023). Our findings build on these previous studies by revealing a direct ethylene-transcription factor-PR gene regulatory axis that sustains the plant immune response during *R. solanacearum* infection.

## Conclusion

This study employed RNA-seq to characterize the root transcriptional profiles of two wilt-resistant tomato cultivars (“ZJ-7” and “04056”) and the susceptible AC following inoculation with *R. solanacearum*. Our results reveal that ethylene serves as a core regulator of resistance, acting to induce the transcription factors SlbHLH25 and SlbHLH25-like, which in turn sustain the expression of *PR* gene during the early stage of infection. In contrast, the susceptibility of AC is attributed to impaired ACS-mediated ethylene biosynthesis, which leads to the abrogation of *SlbHLH25*/*SlbHLH25-like* and *PR* gene expression at 6 hpi. Collectively, these findings elucidate the pivotal role of ethylene in maintaining tomato immune responses against *R. solanacearum* and provide a theoretical framework for the development of molecular markers and genetic engineering strategies aimed at enhancing bacterial wilt resistance in tomato.

## Materials and methods

### Plant materials and growth conditions

Tomato plants (AC, “ZJ-7” and “04056”) were grown in plastic pots on a peat:perlite mixture (2:1, v/v) and kept in a growth room under conditions of 28 °C, 60-70 µmol photons m^-2^ s^-1^, 70% relative humidity and a 16-hour light/8-hour dark photoperiod.

### Treatments of *R. solanacearum* infection

Soil-grown tomato plants were inoculated with a 5 mL cell suspension of the *R. solanacearum* FJAT-91 strain (10^8^ cfu/mL) via root irrigation. The plants were then placed in a growth room with 80% relative air humidity under the aforementioned conditions. The phenotype was observed and the disease index was measured according to **Supplementary Table S3**. Bacterial reproduction and diffusion were calculated using CFU (colony-forming units) after leaf injection treatment with the *R. solanacearum* cell suspension. Briefly, 100 µL of a *R. solanacearum* cell suspension with a concentration of 10^4^ cfu/mL (OD_600_ = 0.4) was injected into the inoculation site of tomato leaves using a disposable syringe. Reactive oxygen species (ROS) bursts and H_2_O_2_ accumulation at pathogen invasion sites were detected in leaves after aforementioned injection with *R. solanacearum*.

### RNA-seq analysis

Total RNA was extracted from tomato roots harvested at various time points after infection with *R. solanacearum* using an MagMAX™-96 Total RNA Isolation Kit (Ambion, AM1830), following the manufacturer’s instructions. Sequencing libraries were constructed with barcodes using a TruSeq RNA Sample Preparation Kit (Illumina), with three biological replicates per sample group, and were sequenced on a DNBSEQ-T7 platform. After removing the low-quality reads, the clean reads were mapped to the tomato genome (SL3.1; https://www.ncbi.nlm.nih.gov/datasets/genome/?taxon=4081) using TopHat v2.0.12 (Trapnell et al., 2009). Gene expression levels were normalised using fragments per kilobase of transcript per million mapped reads (FPKM) (Simon et al., 2015). Differential expression based on the three biological replicates in each group was calculated using DESeq2 (version 1.22.2) (Simon and Wolfgang, 2010). The resulting P values were adjusted to control the false discovery rate (Yoav and Yosef, 1995). Genes with a log_2_ fold change ≥1 and an adjusted p value <0.05 were identified as differentially expressed genes (DEGs). Enrichment analyses of GO terms and KEGG pathways were performed using databases obtained from http://geneontology.org/ and http://www.genome.jp/kegg/, respectively.

### RT-qPCR

RT-qPCR assays were performed as previously described (Cai et al., 2024; Cheng et al., 2024). In brief, total RNA was extracted from tomato roots using an EasyPure^®^ Universal Plant Total RNA Kit (TransGen, ER302-01), then subjected to reverse transcription using HiScript III RT SuperMix (Vazyme Biotech, R323-01). Quantitative PCR (qPCR) was then performed using SYBR Green reagent (Vazyme Biotech, Q312-02), following the manufacturer’s instructions. Four independent biological replicates were performed in total using specific primers (see **Supplementary Table S2**). The collected data were analysed using the method reported by Livak and Schmittgen (Livak & Schmittgen, 2001) and normalised to the expression level of SlACTIN using the 2^-ΔΔCT^ method.

### Trypan blue and DAB staining

The accumulation of intracellular hydrogen peroxide (H_2_O_2_) and reactive oxygen species was visually assessed by staining the leaves collected from tomato plants with 1 mg/mL diaminobenzidine (DAB) or Trypan blue (Papageorgiou et al., 2016). Following an overnight incubation in DAB or trypan blue, the DAB-stained leaves were left to destain in absolute ethanol. Meanwhile, the Trypan blue-stained leaves were boiled in a solution of lactic acid, glycerol and absolute ethanol (at a ratio of 1:1:3, v/v/v), after which they were also left to destain in absolute ethanol.

### Application of ethephon

Application of ethephon were performed as previously described (Waseem et al., 2019). The AC Tomato plants at the 6-leaf stage were sprayed with 0.4% ethephon in sterile ddH_2_O. Control plants were sprayed with sterile ddH_2_O.

### Statistical analyses

Statistical analyses were conducted using the DPS statistical software package. All data are presented as the mean ± standard deviation (SD) derived from three or four independent biological replicates. Significant differences among group means were determined via Fisher’s protected least significant difference (LSD) test, with distinct uppercase letters indicating statistically significant differences at the *p* < 0.01 level.

## Data Availability

The datasets presented in this study can be found in online repositories. The names of the repository/repositories and accession number(s) can be found in the article/**Supplementary Material**.
