# Supplementary material for: Tomato activates ethylene signaling to maintain pathogenesis-related genes expression for conferring bacterial wilt resistance
Source: Front Plant Sci. 2026 Feb 16;16:1753391. doi: 10.3389/fpls.2025.1753391 (PMC12950712; doi:10.3389/fpls.2025.1753391)
Supplement: Supplementary file 4 [file Image1.pdf]

Supplemental Figures

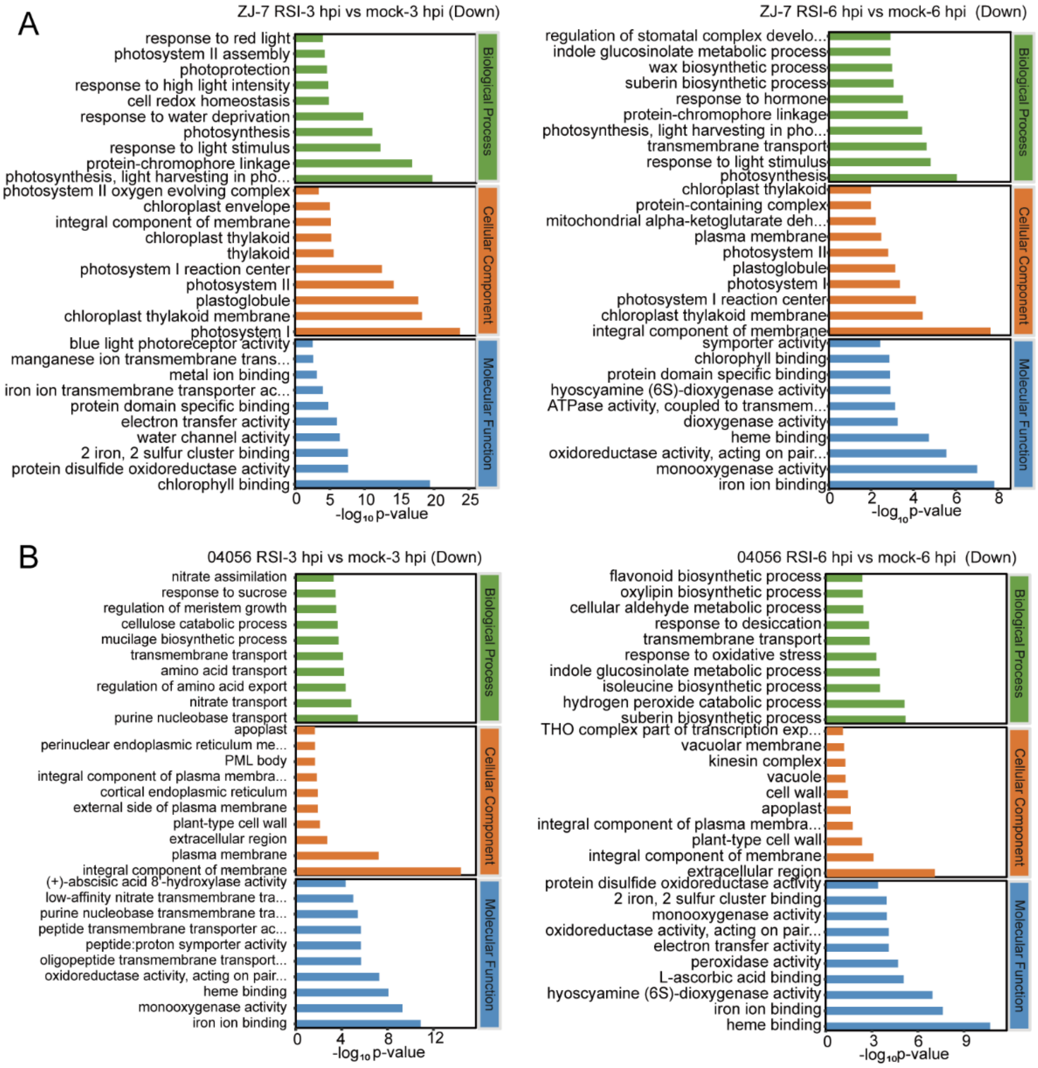

Figure S1 GO enrichment analysis of downregulated DEGs in "ZJ-7" and "04056" up-regulated DEGs identified at 3 and 6 hpi with *R. solanacearum*. (A) Top enriched GO terms of downregulated DEGs in "ZJ-7" at 3 hpi (left panel) and 6 hpi (right panel) post *R. solanacearum* inoulation. (B) Top enriched GO terms of downregulated DEGs in "04056" at 3 (left panel) and 6 (right panel) hpi post *R. solanacearum* inoculation. Mock, normal condition; RSI, *R. solanacearum* infection.

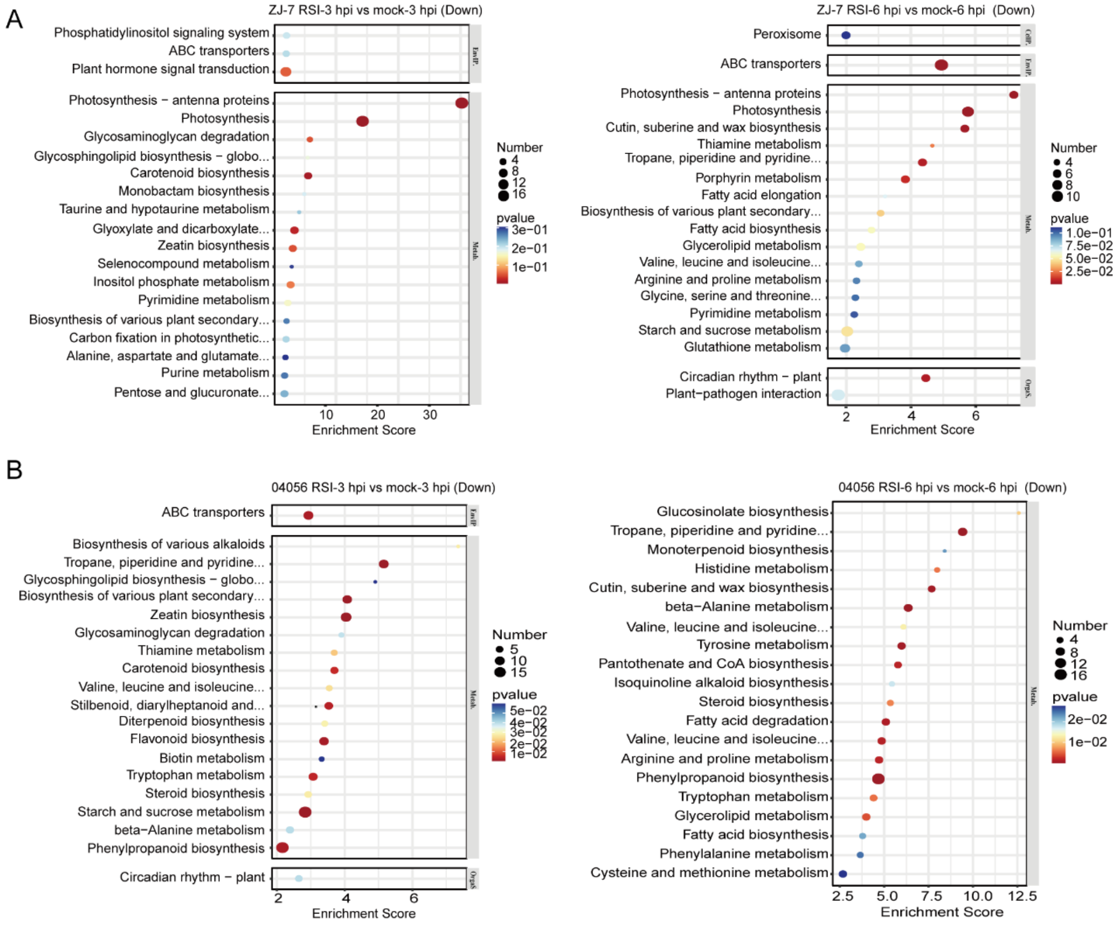

Figure S2 KEGG pathway enrichment analysis of down-regulated DEGs in"ZJ-7" and "04056" at 3 and 6 hpi following *R. solanacearum* infection. (A) Top KEGG pathways significantly enriched in down-regulated DEGs of "ZJ-7" at 3 hpi (left panel) and 6 hpi (right panel) with *R. solanacearum*. (B) Top KEGG pathways significantly enriched in down-regulated DEGs of "04056" at 3 hpi (left panel) and 6 hpi (right panel) with *R. solanacearum*. Mock, normal condition; RSI, *R. solanacearum* infection.
